# Supplementary material for: The Tumorigenicity of Breast Cancer Cells Is Reduced upon Treatment with Small Extracellular Vesicles Isolated from Heparin Treated Cell Cultures
Source: Int J Mol Sci. 2023 Oct 29;24(21):15736. doi: 10.3390/ijms242115736 (PMC10649933; doi:10.3390/ijms242115736)
Supplement: Supplementary file 1 [file ijms-24-15736-s001.zip › ijms-2616365-supplementary.pdf]

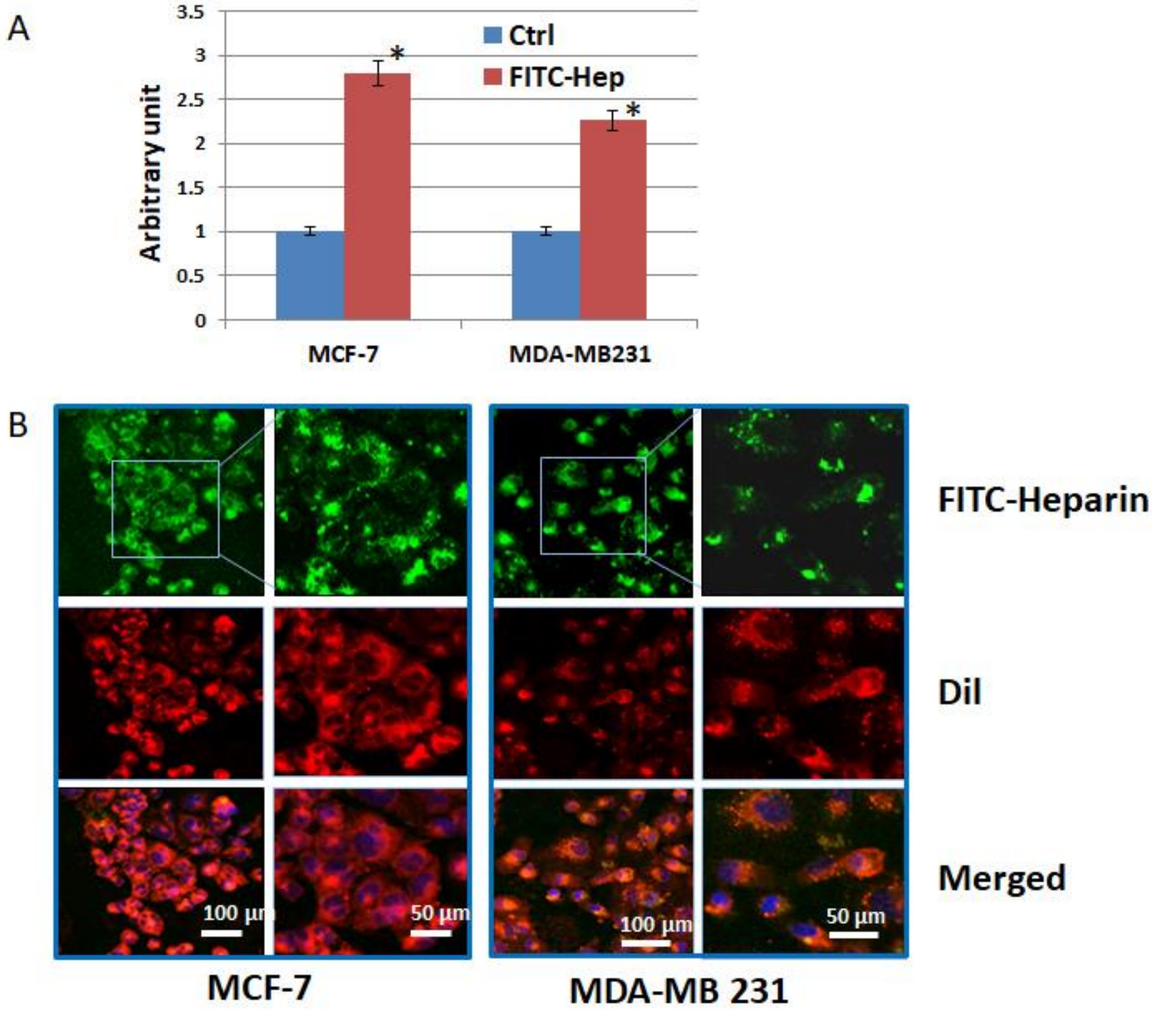

Figure S1. The level of internalization of FITC-Heparin by MCF-7 and MDA-MB231 cells after 1.5-hour incubation. A. Cells were cultured until 70 % confluence in medium containing 15% FBS. The medium was replaced with 1% FCS medium for 4 hours and then the cells changed into fresh 1% FCS medium with or without FITC-heparin (Life Technologies) at a final concentration of 1 µg/ml. After 1.5 hour at RT, the cells were stripped off with 2mM EDTA and the level of intracellular uptake of heparin was analysed by flow cytometry. Graph represents mean fluorescent value (20000 event/sample) of one of three representative experiments ± SD; Values are normalized to control (no FITC-heparin) with each cell line (n=1) and shown as arbitrary units. Asterisks indicate the degree of statistical significance (* $p<0.05$) as determined in comparing with non-FITC-heparin treated group. **B**. Confocal microscopy of cells cultured in 6 well chamber slides and treated with FITC-heparin for 1 hour and then with 0.5 µg/ml Dil (Life Technologies, UK) before the end of incubation following the manufacturer’s instruction. The cells were fixed with 3.7 %

paraformaldehyde in PBS for 10 min while maintained on ice and the slides were mounted in VECTASHIED containing DAPI (Vector Laboratories, UK) before confocal images of samples were obtained with a Leica confocal microscopy (Leica DMI4000B, German).

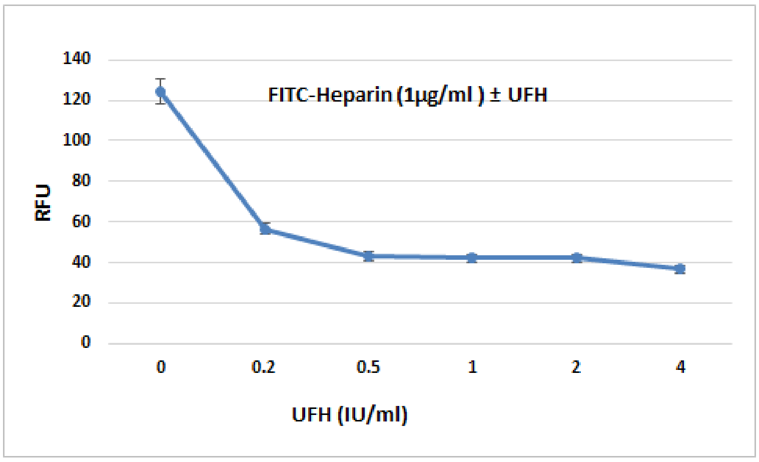

Figure S2. FITC-heparin occurs in sEV separated from conditioned medium of FITC-heparin treated MCF-7 cells.

MCF-7 cells were treated with FITC-Heparin at a final concentration of 1 μg/ml for 2 days alone or in the presence of a serial concentration of UFH (0.2 - 4 IU/ ml). Cells with no added FITC-heparin were used as controls. sEV were isolated using ExoQuik-TC exosome isolation reagents (System Biosciences, UK) from the culture medium of each experimental batch of cells. The assay of FITC-heparin concentration in sEV (2μg of each) was performed using a Molecular Devices fluorescence plate reader (Excitation 485 nm / Emission 538 nm),

It was observed that heparin occurs in sEV prepared from FITC-heparin treated cells. The specificity of the occurrence of FITC–heparin within the contents of the sEV was demonstrated by the progressive reduction in fluorescent label in the presence of increasing concentrations of unlabelled UFH.
